# Thermospermine Is an Evolutionarily Ancestral Phytohormone Required for Organ Development and Stress Responses in *Marchantia Polymorpha*

**DOI:** 10.1093/pcp/pcae002

**Published:** 2024-01-05

**Authors:** Takuya Furumoto, Shohei Yamaoka, Takayuki Kohchi, Hiroyasu Motose, Taku Takahashi

**Affiliations:** Department of Biological Science, Graduate School of Environmental, Life, Natural Science and Technology, Okayama University, Tsushimanaka 3-1-1, Okayama, 700-8530 Japan; Graduate School of Biostudies, Kyoto University, Kyoto, 606-8502 Japan; Graduate School of Biostudies, Kyoto University, Kyoto, 606-8502 Japan; Department of Biological Science, Graduate School of Environmental, Life, Natural Science and Technology, Okayama University, Tsushimanaka 3-1-1, Okayama, 700-8530 Japan; Department of Biological Science, Graduate School of Environmental, Life, Natural Science and Technology, Okayama University, Tsushimanaka 3-1-1, Okayama, 700-8530 Japan

**Keywords:** ACAULIS5, *Marchantia polymorpha*, Polyamine, Sexual organ, Stress response, Thermospermine

## Abstract

Thermospermine suppresses auxin-inducible xylem differentiation, whereas its structural isomer, spermine, is involved in stress responses in angiosperms. The thermospermine synthase, ACAULIS5 (ACL5), is conserved from algae to land plants, but its physiological functions remain elusive in non-vascular plants. Here, we focused on Mp*ACL5*, a gene in the liverwort *Marchantia polymorpha*, that rescued the dwarf phenotype of the *acl5* mutant in *Arabidopsis*. In the Mp*acl5* mutants generated by genome editing, severe growth retardation was observed in the vegetative organ, thallus, and the sexual reproductive organ, gametangiophore. The mutant gametangiophores exhibited remarkable morphological defects such as short stalks, fasciation and indeterminate growth. Two gametangiophores fused together, and new gametangiophores were often initiated from the old ones. Furthermore, Mp*acl5* showed altered responses to heat and salt stresses. Given the absence of spermine in bryophytes, these results suggest that thermospermine has a dual primordial function in organ development and stress responses in *M. polymorpha*. The stress response function may have eventually been assigned to spermine during land plant evolution.

## Introduction

Polyamines are low-molecular-weight aliphatic compounds with multiple amino groups. They are involved in various biological activities including stabilization of nucleic acids, mRNA translation and modulation of protein functions (Igarashi and Kashiwagi 2010, Pegg 2016). Ubiquitous polyamines, putrescine (diamine) and spermidine (triamine), are essential for all organisms. Tetramine, spermine, is generally found in animals and fungi but not always in bacteria and plants (Takano et al. 2012). Currently, no spermine synthase genes have been identified in mosses or ferns. Spermine synthase is an aminopropyl transferase that mediates the synthesis of spermine from spermidine and an aminopropyl moiety provided by decarboxylated *S*-adenosylmethionine. In *Arabidopsis*, a loss-of-function mutant of a single gene for spermine synthase shows no morphological phenotype under normal growth conditions (Imai et al. 2004). However, there is increasing evidence that spermine is implicated in the response to biotic and abiotic stresses in angiosperms (Tiburcio et al. 2014).

Thermospermine is a structural isomer of spermine that was first found in an extremely thermophilic bacterium (Oshima 1979). In contrast to spermine, thermospermine is widespread in the plant kingdom. The gene encoding thermospermine synthase, which was identified from its mutant, *acaulis5* (*acl5*), in *Arabidopsis* (Hanzawa et al. 1997) and named *ACL5*, may have been acquired early in plant evolution by horizontal gene transfer from bacteria (Minguet et al. 2008). *ACL5* is exclusively expressed in xylem precursor cells and is involved in suppressing excessive xylem formation (Clay and Nelson 2005). The excess xylem phenotype associated with the dwarfism of *acl5* is partially restored by exogenous thermospermine (Kakehi et al. 2008). Isolation and analysis of *suppressor of acl5* (*sac*) mutants revealed that thermospermine enhances the mRNA translation of *SAC51*, which encodes a basic helix-loop-helix (bHLH) protein and plays a role in suppressing xylem differentiation (Imai et al. 2006). Thermospermine may release the *SAC51* mRNA from an inhibitory effect of its own conserved upstream open reading frame (uORF) on the main ORF translation, but the precise mode of action remains unknown (Imai et al. 2006, Kakehi et al. 2015, Cai et al. 2016, Ishitsuka et al. 2019). Furthermore, a series of genetic analyses have shown the involvement of the *SAC51* family in thermospermine-mediated negative feedback regulation of auxin-inducible xylem differentiation (Katayama et al. 2015, Vera-Sirera et al. 2015). Auxin promotes the expression of *TARGET OF MONOPTEROS 5* (*TMO5*), which encodes a bHLH transcription factor that forms a heterodimer with another bHLH transcription factor, LONESOME HIGHWAY (LHW). The TMO5-LHW complex promotes xylem proliferation and induces the expression of *ACL5* and *SACL3. SACL3* is a member of the *SAC51* family whose product competes with TMO5 for the binding with LHW to suppress excess xylem formation (Katayama et al. 2015, Vera-Sirera et al. 2015).

Bryophytes, including liverworts and mosses, do not develop vascular systems but have genes highly homologous to *ACL5*, which suggests that there may be as-yet-unidentified function of thermospermine in the basal land plants (Solé-Gil et al. 2019). Thus, we focused on Mp*ACL5*, the only *ACL5* gene in the liverwort *Marchantia polymorpha*. In this study, we generated loss-of-function mutants of Mp*ACL5.* Characterization of the mutants revealed that thermospermine is critically required for both organ development and stress responses in *M. polymorpha*.

## Results

### Mp*ACL5* is preferentially expressed in meristematic regions and reproductive organs

**Fig. 1:**
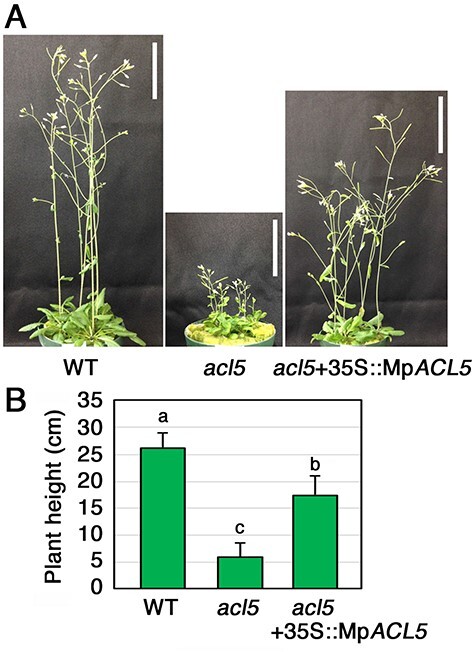
Complementation of the *Arabidopsis acl5* phenotype by Mp*ACL5*. (A) Phenotypes of wild-type Col-0 (wild type, (WT)), *acl5-1* and a representative transgenic line of *acl5-1* expressing Mp*ACL5* under the CaMV 35S promoter. Plants were grown for 5 weeks on rockwool placed in vermiculite. Bars = 5 cm. (B) Plant height of 30-day-old plants of the wild-type Col-0, *acl5-1* and *acl5-1* with the 35S:Mp*ACL5*. Bars indicate SD (*n* = 10). The different letters indicate significant differences by Tukey’s honestly significant difference (HSD) test (*P* < 0.05).

The Mp*ACL5* gene consists of nine exons and eight introns whose positions are conserved in land plants. This gene encodes a protein with 340 amino acid residues with a molecular mass of 37.7 kDa. MpACL5 protein is 63% identical to *Arabidopsis* ACL5 (**Supplementary Fig. S1**). The MpACL5 protein has been shown to function as a thermospermine synthase in yeast cells (Solé-Gil et al. 2019). We detected thermospermine in the extract of *Escherichia coli* that expressed recombinant MpACL5 or AtACL5 (**Supplementary Fig. S2A**). AtACL5 has proven to be a thermospermine synthase both in vitro and in planta (Knott et al. 2007, Kakehi et al. 2008). Thus, we concluded that MpACL5 can also synthesize thermospermine. To determine whether MpACL5 functions as a thermospermine synthase in planta, we generated transgenic *Arabidopsis* lines carrying the Mp*ACL5* cDNA driven by the constitutive cauliflower mosaic virus (CaMV) 35S promoter in the *acl5* mutant background and confirmed that the dwarf phenotype of *acl5* was rescued (**Fig. 1**).

**Fig. 2:**
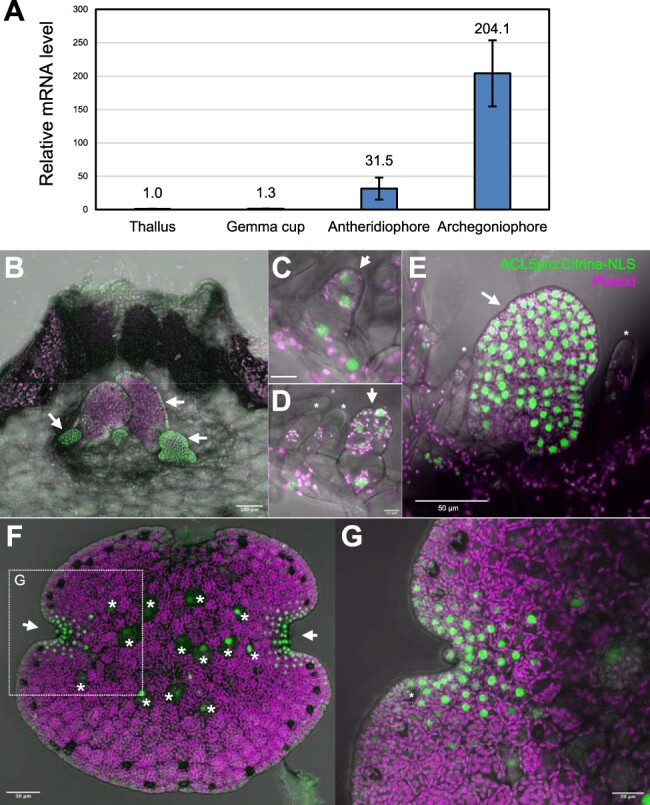
Expression patterns of Mp*ACL5*. (A) RT-qPCR analysis of Mp*ACL5* in thallus, gemma cup and gametangiophore. Bars indicate SD (*n* = 3 of thalli and gemma cups, *n* = 4 of antheridiophores and archegoniophores). (B–G) Citrine-NLS expression under the Mp*ACL5* promoter in confocal *z*-stack images. The autofluorescence of plastids is shown in magenta. (B) A longitudinal section of a gemma cup. Arrows indicate gemmae. (C–E) Developing gemmae [early stage in (C) and (D)]. Arrows and asterisks indicate gemmae and mucilage papillae, respectively. (F) A matured gemma. Arrows and asterisks indicate apical notches and rhizoid cells, respectively. (G) An enlarged view of the boxed region in (F).

**Fig. 3:**
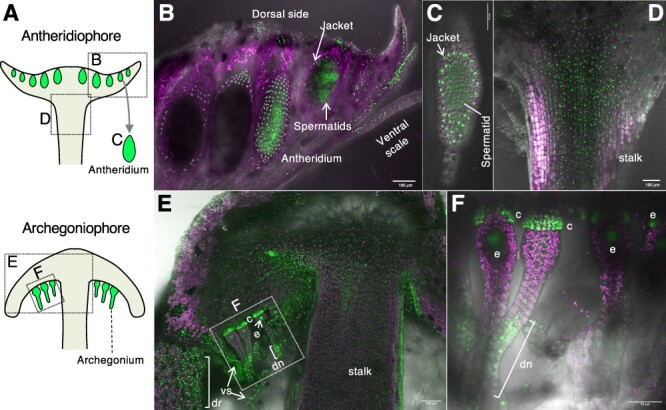
Promoter activity of Mp*ACL5* in gametangiophores. Citrine-NLS expression under the Mp*ACL5* promoter in confocal *z*-stack images. The autofluorescence of plastids is shown in magenta. (A) Schematic diagrams of gametangiophores. (B) A longitudinal section of an antheridiophore. (C) An isolated antheridium. (D) A longitudinal section of the apical region of a stalk in an antheridiophore. (E) A longitudinal section of an archegoniophore. (F) A magnified view of the boxed region in (E). c, collar; dn, distal neck; dr, digitate ray; e, egg cell; vs, ventral scale.

Reverse transcription quantitative PCR (RT-qPCR) experiments revealed that the Mp*ACL5* transcript was detected in all organs tested and preferentially accumulated in both the antheridiophores and archegoniophores, the male and female reproductive organs (**Fig. 2A**). We also generated transgenic *M. polymorpha* lines that expressed Citrine with a nuclear localization signal (Citrine-NLS) under the control of the Mp*ACL5* promoter. Intense Citrine-NLS fluorescence was observed in the developing gemmae (**Fig. 2B****–****E**) and bilateral apical notches of the matured gemma (**Fig. 2F**, **G**). The Mp*ACL5* promoter was active in the early developmental stages of gemmae (**Fig. 2C**, **D**) and rhizoids (**Fig. 2F**). Intense Citrine-NLS fluorescence was also observed in antheridiophores and archegoniophores, reproductive organs (**Fig. 3**). In the antheridiophores, promoter activity was evident in the jacket cells and spermatids of the antheridia (**Fig. 3B**, **C**). Citrine-NLS fluorescence was also detected in the ventral scales, dorsal cells and the apical region of the stalks (**Fig. 3B**, **D**). In the archegoniophores, the promoter activity was conspicuous in collar cells and distal neck cells of the archegonia. Fluorescence was also detected in the egg cells, ventral scales, digitate rays and apical stalk cells (**Fig. 3E**, **F**). These results suggest that Mp*ACL5* is involved in cell proliferation, meristem maintenance and reproductive development.

### Mp*acl5* mutants show abnormal growth

**Fig. 4:**
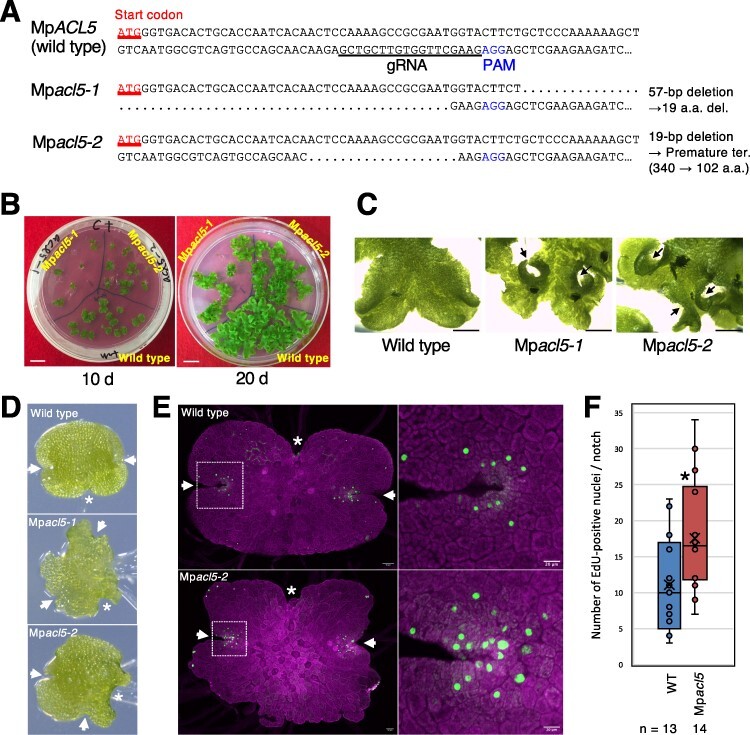
Generation of Mp*acl5* mutants. (A) DNA sequences of Mp*ACL5* around the CAS9 target site. Dots indicate nucleotides deleted by genome editing. (B) Wild-type (Tak-1) and Mp*acl5* mutant thalli grown on B5 agar media for 10 and 20 d. Bars = 1 cm. (C) Magnified views of wild-type (Tak-1) and Mp*acl5* mutant thalli grown on B5 agar media for 20 d. Arrows indicate curled thalli. Bars = 1 mm. (D) Morphology of 1-day-old gemmalings. Arrows and asterisks indicate apical notches and traces of stalks, respectively. (E) Three-day-old gemmalings of the wild type (Tak-1) and Mp*acl5-2* stained by EdU. The right panels are magnified views of the boxed regions in the left panels. Green, EdU-positive nuclei; magenta, autofluorescence of plastids. Arrows and asterisks indicate apical notches and traces of stalks, respectively. (F) Number of EdU-positive nuclei. Data are shown in the box plot (*n* = number of notches). The bottom and top edges of the box are 25th and 75th percentiles, respectively. The horizontal line and cross in the box represent the median and mean value, respectively. The whiskers range from the minimum to the maximum values. An asterisk indicates a significant difference (Student’s *t* test, *P* < 0.04).

Loss-of-function mutants of Mp*ACL5* were generated by the CRISPR/Cas9 genome editing system to explore the function of Mp*ACL5* (Sugano et al. 2018). The guide RNA was designed from the 95th to 112th bases of the Mp*ACL5* coding sequence (**Fig. 4A**), which corresponds to the N-terminal region essential for the binding of substrates (spermidine and decarboxylated *S*-adenosylmethionine) and its catalytic activity. We isolated two independent mutant lines, Mp*acl5-1* and Mp*acl5-2*. Mp*acl5-1* harbors a 57-bp deletion, which causes the deletion of 19 amino acid residues, including Lys-31, essential for binding decarboxylated *S*-adenosylmethionine. This line was later found to be a female line. Mp*acl5-2* is a male line with a 19-bp deletion, which results in a frameshift mutation, creating a premature stop codon and a putative truncated peptide of 102 amino acid residues. Thus, both Mp*acl5-1* and Mp*acl5-2* may represent loss-of-function alleles. We confirmed that Mp*acl5* mutants have no detectable levels of thermospermine (**Supplementary Fig. S2B**).

Both mutant lines exhibited similar morphological defects in flat, leaf-like vegetative organs, known as thalli, which grow and periodically bifurcate through activity in the apical notch (**Fig. 4B**). Thalli exhibit a clear dorsal-ventral polarity. Air pores, photosynthetic assimilatory filaments and gemma cups are formed on the dorsal side, while rhizoids and ventral scales are generated on the ventral side. Rhizoids elongate by tip growth to form hair-like protrusions. In Mp*acl5* mutants, thallus growth was severely retarded, and the peripheral region near the apical notch was curly and distorted toward the outside (**Fig. 4C**). In the early stages of the gemmalings, the wild type had bilateral apical notches, smooth thallus margins and rounded lobes, whereas the Mp*acl5* mutants showed altered positions of notches, serrated margins and irregularly expanded lobes (**Fig. 4D**). We further analyzed the morphology of the apical notches and their cell proliferation activity by 5-ethynyl-2'-deoxyuridine (EdU) staining (**Fig. 4D, E**). There were more EdU-positive cells in Mp*acl5*, but the positive cells were localized in the apical notches in both the wild type and Mp*acl5*. There were also more EdU-positive cells in mature thalli and early reproductive organs in Mp*acl5* compared to the wild type (**Supplementary Fig. S3**). On the other hand, no morphological abnormalities were observed in the gemma cups, air pores, assimilatory filaments, ventral scales or rhizoids.

**Fig. 5:**
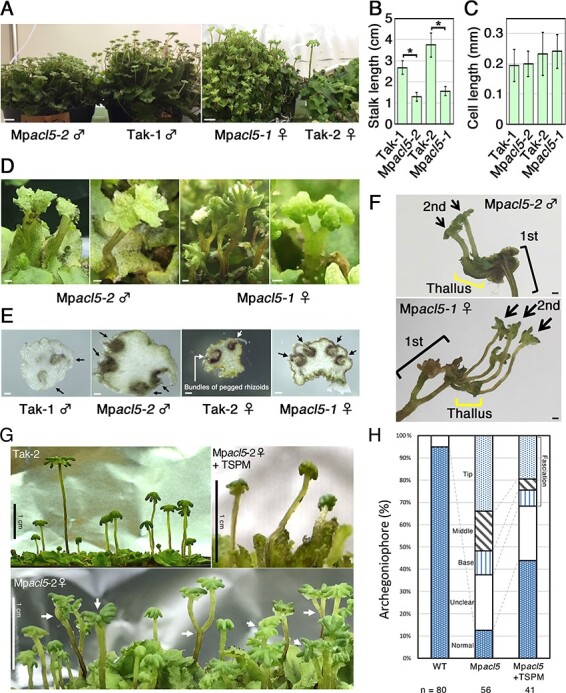
Phenotype of sexual organs in Mp*acl5* mutants. (A) Gross morphology of sexual organs in the wild type and Mp*acl5* mutants. Bars = 1 cm. (B) Length of stalks of sexual organs in the wild type and Mp*acl5* mutants. Bars indicate SD (*n* = 20). Asterisks indicate values determined by Student’s *t* test to be significantly different from the wild type (*P < 0.05). (C) Length of stalk cells of sexual organs in the wild type and Mp*acl5* mutants. Bars indicate SD (*n* = 20 cells from four stalks). (D) Fasciation phenotype of stalks in Mp*acl5* mutants. Bars = 1 mm. (E) Cross-sections of stalks of sexual organs in the wild type and Mp*acl5* mutants. Two bundles of pegged rhizoids (arrows) were observed in the wild type, while four bundles were formed in Mp*acl5*. Bars = 100 µm. (F) Secondary sexual organs formed in Mp*acl5*. Secondary sexual branches were frequently formed from primary branches in both male and female Mp*acl5* mutants. Bars = 1 mm. (G) Fasciation phenotype of Mp*acl5-2* female plants in the F1 progeny obtained by the crossing of the wild-type Tak-2 with Mp*acl5-2.* The fasciation was recovered by the daily application with 100 µM thermospermine (upper right panel). Arrows indicate fasciated archegoniophores. (H) Quantification of the fasciation phenotype of archegoniophores of Tak-2 (WT), female F1 plants harboring the Mp*acl5-1* or Mp*acl5-2* mutation (Mp*acl5*) and Mp*acl5* mutants daily applied with 100 µM thermospermine. The fasciation phenotype was classified into three coalescence patterns depending on where they branch; two gametangiophores were branched at the base of the stalk (base), in the middle of the stalk (middle) or at the tip of the stalk (tip). Two fasciated archegoniophores were counted as one archegoniophore. Some archegoniophores were too small to be determined whether they were coalesced (unclear).

**Fig. 6:**
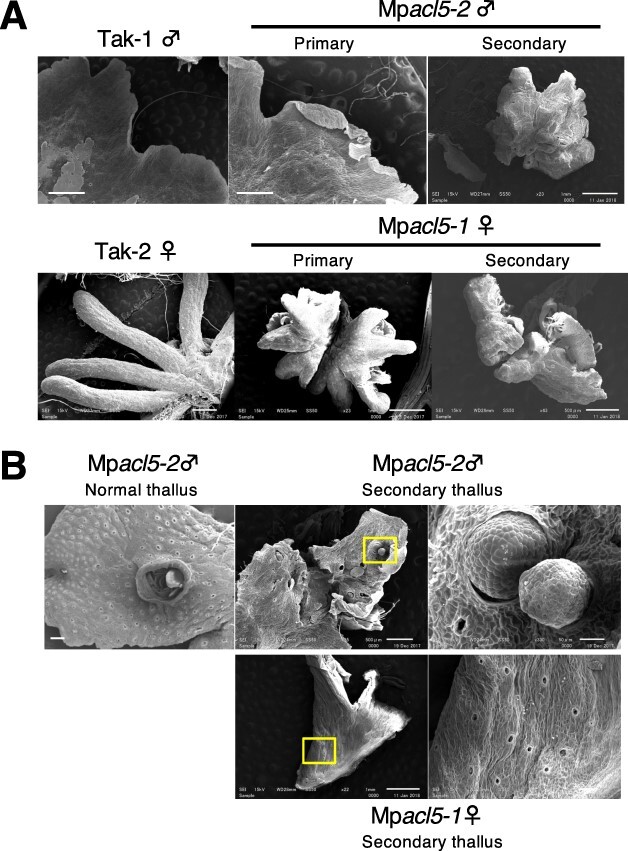
SEM images of gametangiophores and thalli in the wild type and Mp*acl5* mutants. (A) Morphology of antheridiophores and archegoniophores. Bars = 0.5 mm (upper left, upper center, lower right), 1 mm (upper right, lower left, lower center). (B) Morphology of a normal thallus and the secondary thalli generated from the primary branches in Mp*acl5*. Antheridia were formed on the secondary thallus of Mp*acl5*. Bars = 1 mm (upper left, lower left), 0.5 mm (upper center), 50 µm (upper right), 100 µm (lower right).

Then, we successfully induced archegoniophores in Mp*acl5-1* and antheridiophores in Mp*acl5-2*, indicating that MpACL5 is not essential for the phase transition from vegetative to reproductive growth. However, both mutants produced shorter gametangiophore stalks than the wild type (**Fig. 5A, B**). There was no significant difference in cell length between the wild type and Mp*acl5* mutants, suggesting that the short stalk phenotype is caused by reduced cell number (**Fig. 5C**). Furthermore, the mutant stalks exhibited a fasciation phenotype. In the wild type, one gametangiophore usually develops from an apical notch, but in Mp*acl5* mutants, two gametangiophores were formed from an apical notch (**Fig. 5D**). Three coalescence patterns were observed depending on where they branched; two gametangiophores were branched at the base of the stalk, in the middle of the stalk or at the tip of the stalk. Although the fasciation phenotype was observed in both male and female gametangiophores of Mp*acl5*, the fasciation of archegoniophores was more prominent than that of antheridiophores; the former showed three coalescence patterns, whereas the mutant antheridiophores had stalks that branched at the tip. Moreover, while two bundles of pegged rhizoids formed at the stalk of the wild-type gametangiophores, four bundles of pegged rhizoids were found in both male and female gametangiophores of Mp*acl5* mutants (**Fig. 5E**), indicating a fusion of stalks. Interestingly, the gametangiophores of Mp*acl5* mutants often initiated secondary thalli from their peripheral tip region and generated secondary gametangiophores, resulting in double-decker gametangiophores (**Fig. 5F**). Under the scanning electron microscope (SEM), the mutant gametangiophores showed a distorted and rough surface morphology. Digitate rays of archegoniophores were smaller in the mutants than in the wild type. The size reduction and the morphological defects were enhanced in the secondary gametangiophores. The secondary thalli did not form gemma cups. The male secondary thalli generated antheridia on the dorsal side, some of which were extended from the antheridia cavities (**Fig. 6**). Thus, the secondary thallus may have characteristics of both vegetative and reproductive organs.

To determine whether the fasciation phenotype is a result of the Mp*ACL5* mutations or the potential second-site mutations, we introgressed the Mp*acl5-1* (female) and Mp*acl5-2* (male) alleles into Takaragaike-1 (Tak-1) (male) and Takaragaike-2 (Tak-2) (female), respectively, by sexual crossing. All 16 F1 plants that harbored the Mp*acl5-1* or Mp*acl5-2* mutation exhibited the fascination in the gametangiophores (**Fig. 5G**, **Supplementary Fig. S4**, showing the male Mp*acl5-1* and female Mp*acl5-2* mutant). Because the fasciation phenotype was the most prominent in archegoniophores, we quantitatively analyzed the morphology of archegoniophores (**Fig. 5H**). Fasciation was observed in more than 60% of archegoniophores, about half of which were branched at the tip of the stalks. When thermospermine solutions (100 µM) were applied as droplets to the apices of archegoniophores, the fasciation phenotype was partially suppressed (**Fig. 5G, H**). These results indicate that the loss of thermospermine caused the fasciation phenotype of reproductive organs in *M. polymorpha*.

### Mp*BHLH42* may not be a target of thermospermine

Only a single homolog to the *Arabidopsis SAC51* was identified in the *M. polymorpha* genome (MpBHLH42, Mp5g09710 and Mapoly0048s0099). Translation of the *SAC51* family genes is the only known process targeted by thermospermine. Although the 5ʹ leader sequence of the Mp*BHLH42* mRNA contains seven AUGs (**Supplementary Fig. S5A**), none of the three long uORF-encoded peptides are homologous to those conserved among the SAC51 family in vascular plants (**Supplementary Fig. S5B**). We also found that the 5ʹ leader showed no response to thermospermine when it was fused to the β-glucuronidase reporter gene and expressed under the CaMV 35S promoter in transgenic *Arabidopsis* plants (**Supplementary Fig. S5C**). Thus, *M. polymorpha* may have different molecular mechanisms downstream of thermospermine compared to those in *Arabidopsis*.

### Mp*acl5* mutants show altered responses to stresses

**Fig. 7:**
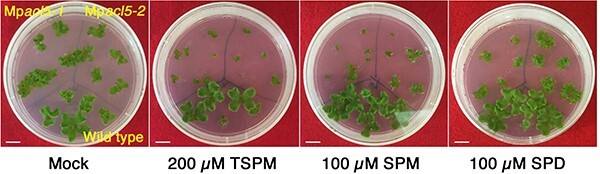
Effect of polyamines on the growth of wild-type (Tak-1) and Mp*acl5* mutant thalli. Gemmae were planted on B5 agar media supplemented with thermospermine (TSPM), spermine (SPM) or spermidine (SPD) and grown for 20 d. Bars = 1 cm.

To examine whether exogenous thermospermine can rescue the growth defects of thalli in Mp*acl5*, thalli were grown in media supplemented with thermospermine or spermidine, a precursor of thermospermine (**Fig. 7**). Unexpectedly, both thermospermine and spermidine severely suppressed the growth of the thalli in Mp*acl5* but not in the wild type. Spermine was also inhibitory to the growth only in Mp*acl5*. These inhibitory effects contrasted with the recovery of the *acl5* mutant of *A. thaliana* by exogenous thermospermine (Kakehi et al. 2008). The hypersensitivity of Mp*acl5* mutants to exogenous polyamines suggests a profound effect of thermospermine deficiency on cellular physiology in *M. polymorpha*.

**Fig. 8:**
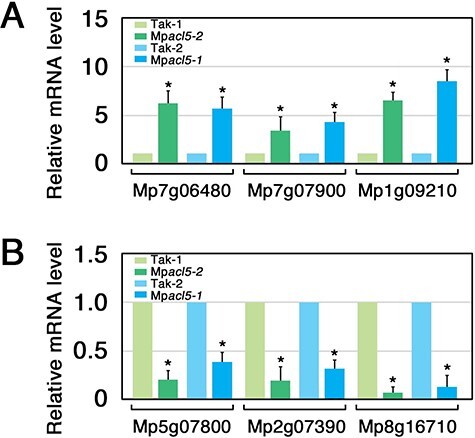
The mRNA levels of representative differentially expressed genes in Mp*acl5*. RT-qPCR analysis was conducted to confirm the RNA-seq data, which showed a set of genes upregulated or downregulated in the Mp*acl5* mutant thalli. Three representative genes whose expression levels were increased (A) and decreased (B) in Mp*acl5* are shown. Mp7g06480 and Mp7g07900 encode small heat shock proteins. Mp1g09210 and Mp8g16710 encode proteins with unknown function. Mp5g07800 and Mp2g07390 encode fucosidase-like protein and caffeic acid 3-*O*-methyltransferase-like protein, respectively. Bars indicate SD (*n* = 3). Asterisks indicate values determined by Student’s *t* test to be significantly different from the wild type (*P < 0.05).

To examine the effects of Mp*acl5* mutations on gene expression profiles, we performed RNA-seq analyses of wild type and Mp*acl5* thalli and found a large number of genes with altered expression levels in the mutants (**Supplementary Table S1**). To confirm the RNA-seq data, we subsequently performed RT-qPCR experiments for the selected genes and found that the expression of small heat shock protein (HSP) genes, Mp7g06480 (Mapoly0057s0019) and Mp7g07900 (Mapoly0076s0004), and a gene with unknown function, Mp1g09210 (Mapoly0036s0157), remarkably increased while genes encoding putative fucosidase-like protein (Mp5g07800 and Mapoly0127s0004), caffeic acid 3-*O*-methyltransferase-like protein, (Mp2g07390, Mapoly0015s0026 and MpOMT8) and an unknown protein (Mp8g16710 and Mapoly0030s0004) were downregulated in Mp*acl5* (**Fig. 8**).

**Fig. 9:**
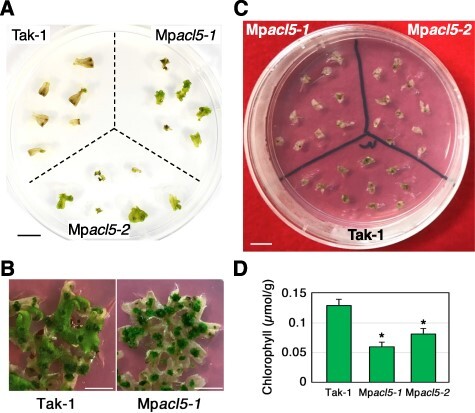
Altered responses of Mp*acl5* mutants to heat and salt stress. (A, B) Effect of heat stress on the growth of the wild type and Mp*acl5* mutants. (A) Gemmalings of the wild type and Mp*acl5* mutants were grown for 2 weeks on the B5 agar medium at 22°C, treated at 37°C for 18 h and grown for 4 d at 22°C. Bar = 1 cm. (B) Gemmalings of the wild type and Mp*acl5* mutants were grown for 4 weeks on the B5 agar medium at 22°C, treated at 37°C for 6 h and grown for 5 d at 22°C. Bars = 1 cm. (C) Effect of NaCl on the growth of the wild type and Mp*acl5* mutants. Gemmalings of the wild type and Mp*acl5* mutants were grown for 7 d on the B5 agar medium without NaCl, transferred to the medium supplemented with 200 mM NaCl and grown for 12 d. Bar = 1 cm. (D) Chlorophyll content in the wild type and Mp*acl5* mutants grown in the presence of 200 mM NaCl as in (C). Bars indicate SD (*n* = 3). Asterisks indicate values determined by Student’s *t* test to be significantly different from the wild type, Tak-1 (*P < 0.05).

We then analyzed heat stress response in Mp*acl5*. Two-week-old thalli were treated at 37°C for 18 h and further grown at 22°C for 4 d. While the wild-type thalli died, the mutant thalli survived (**Fig. 9A**). On the other hand, when 1-month-old thalli were treated at 37°C for 6 h and further grown at 22°C for 5 d, the wild-type thalli still survived and continued to grow, but the mutant thalli showed severe chlorosis and eventually died (**Fig. 9B**). These results and the absence of spermine synthase genes in bryophytes led us to hypothesize that thermospermine is involved in stress tolerance in *M. polymorpha*. Thus, we examined the salt sensitivity of Mp*acl5*. When grown on media supplemented with 200 mM NaCl, the thalli of the Mp*acl5* mutants showed more severer chlorosis compared to those of the wild type (**Fig. 9C, D**), suggesting that thermospermine is necessary for salt stress tolerance.

## Discussion

In *Arabidopsis* and most likely other vascular plants, *ACL5* expression is induced by auxin and thermospermine is a key signal for avoiding excessive xylem development in the negative feedback system of auxin-dependent vascular formation. In bryophytes, the role of thermospermine has been unknown. Here, we generated knockout mutants of Mp*ACL5*. Mp*ACL5* is preferentially expressed in the apical notches of vegetative thalli and reproductive organs. In accordance with the expression pattern, Mp*acl5* mutants show growth suppression in vegetative thalli and reproductive organs rather than defects of tissue and cellular differentiation (**Figs. 4**, **5**). Thus, thermospermine may promote overall organ growth in the basal land plant, as opposed to its role as a suppressor of xylem development in angiosperms. The increased EdU-positive cells in Mp*acl5* mutants (**Fig. 4**) may be attributed to the increased S-phase duration and/or related to the constitutive stress responses (see later).

The gametangiophores of Mp*acl5* mutants had short stalks and an increased number of bundles of pegged rhizoids, which are thought to serve as a conductive tissue (**Fig. 4**). These morphological defects are reminiscent of the *Arabidopsis acl5* mutant, which has short inflorescence stems with excess xylem. However, we must be cautious about this morphological similarity because these defects are caused by different mechanisms. The dwarf phenotype of gametangiophores is due to reduced cell numbers rather than reduced cell elongation, which is observed in *Arabidopsis acl5* (Hanzawa et al. 1997). The increased rhizoid bundles are a result of the fasciation of two gametangiophores rather than the excess formation of rhizoids (**Fig. 5**). Thus, it would be more appropriate to conclude that thermospermine may promote stalk elongation and suppress the fasciation of gametangiophores. In the latter function, thermospermine may limit initiation and/or proliferation of gametangiophore cells to form a single gametangiophore from an apical notch since two gametangiophores were generated from one apical notch in Mp*acl5* mutants. This could be correlated with another function of thermospermine to suppress indeterminate growth of gametangiophores.

According to previous anatomical studies, gametangiophores retain morphological characteristics of thalli and may form through its extension (Shimamura 2016). In Mp*acl5* mutants, some parts of the gametangiophores appeared to transdifferentiate into vegetative thalli, which in turn developed secondary gametangiophores (**Fig. 6**). In summary, thermospermine may have two distinct functions. One function is to promote the growth of thalli and stalks, and the other is to suppress gametangiophore formation and transdifferentiation of gametangiophores into thalli.

Recent studies indicate that thermospermine is involved in stress tolerance in angiosperms, in addition to spermine (Sagor et al. 2012, Marina et al. 2013, Mo et al. 2015, Zarza et al. 2017). The *Arabidopsis acl5* mutant shows high salt sensitivity caused by increased xylem formation and salt uptake (Shinohara et al. 2019). Similar to those phenotypes in *Arabidopsis*, Mp*acl5* mutants are also susceptible to salt stress (**Fig. 9**). However, the gametophytes of *M. polymorpha* have no internal conductive tissues (Shimamura 2016), so the absorption and transport of solutes are most likely done through intercellular space rather than specific conductive tissues. Thus, the hypersensitivity to salt stress could be attributed to the reduced salt stress tolerance of Mp*acl5* mutants rather than the increased uptake of salt. Taking the increased expression of *HSP* genes into account, deficiency of thermospermine may constitutively stimulate stress response pathways in Mp*acl5* mutants. This possibility does not exclude the direct functions of thermospermine in conferring tolerance to harsh environments such as those proposed in thermophilic bacteria containing thermospermine and various uncommon polyamines (Sakamoto et al. 2022).

Our genetic analyses of Mp*ACL5* revealed the fundamental functions of thermospermine in both organ development and stress tolerances in a basally diverging land plant species. Bryophytes do not contain spermine and the spermine synthase (SPMS) gene. Phylogenetic analyses imply that SPMS may have been acquired from spermatophytes during land plant evolution (Minguet et al. 2008). In this scenario, it is possible that spermine took over the ancient function of thermospermine in stress responses and then thermospermine was co-opted for specific regulation of the development and differentiation of cells and tissues. Thus, our study implies the evolutionarily ancient multiple functions of thermospermine acquired for adaptation to the terrestrial environment during land plant evolution, namely, survival and reproductive success through vigorous growth and stress responses in the harsh environments. Further investigation in algae and other basal land plants will provide insight into the functional origin and diversity of this primordial phytohormone.

## Materials and Methods

### Plant material and growth conditions

*Marchantia polymorpha* accessions Tak-1 (male) and Tak-2 (female) were used as the wild type (Bowman et al. 2017). Thalli, gemmalings and sporelings were grown on the half-strength Gamborg’s B5 medium solidified with 1% agar at 22°C under continuous white light. Gametangiophores were induced under continuous white light with far-red irradiation (Chiyoda et al. 2008). For polyamine treatment, spermidine, spermine and thermospermine (Santa Cruz Biotechnology, Dallas, TX, USA) were dissolved in water and added to the B5 medium at the concentrations of 100 or 200 µM. Dormant gemmae were planted on the B5 medium supplemented with each polyamine.

For *Arabidopsis* experiments, Columbia-0 (Col-0) accession was used as the wild type. Plants were grown on rockwool as described previously (Hanzawa et al. 1997).

### Complementation of *Arabidopsis acl5-1*

The full-length Mp*ACL5* amplified from Tak-1 cDNA by PCR using TaKaRa EX Taq (Takara, Shiga, Japan) with the primers, FXb (TCTAG AATGG GTGAC ACTGC ACCA) and RBm (GGATC CTAAT GGGAT TTCGC ATTGG), was cloned into the pGEM-T Easy cloning vector (Promega, Madison, WI, USA). The fragment was then excised by *Xba*I and *Bam*HI and inserted into the downstream of the CaMV35S promoter in pBI121 (Takara) whose GUS reporter gene was removed in advance to generate the *CaMV35Spro:*Mp*ACL5* construct. *Arabidopsis acl5-1* mutant in the Col-0 background (Hanzawa et al. 1997) was infected with the *Rhizobium tumefaciens* GV3101 (pMP90) strain containing the plasmid to introduce the CaMV35Spro:MpACL5 by the floral dip method (Clough and Bent 1998). Transformants were selected with 40 µg ml^−1^ kanamycin and 100 µg ml^−1^ cefotaxime, and their homozygous T3 progenies were analyzed in further experiments.

### Generation of reporter lines

To construct Mp*ACL5pro:Citrine-NLS*, the Mp*ACL5* genomic region, including a 3324-bp region upstream of the initiation codon and a 24-bp coding region corresponding to an 8-amino-acid segment, was amplified from Tak-1 genomic DNA by PCR using KOD plus (Toyobo, Osaka, Japan) with the primers, MpACL5pro-F-3k (CACC GAT ACA CGG CTC ATG TTG AAA ATT AG) and MpACL5-R-8aa (TGT GAT TGG TGC AGT GTC ACC CAT), and cloned into pENTR/D-TOPO (Thermo Fisher Scientific, Waltham, MA, USA). This entry vector was used in the LR reaction with the Gateway binary vector pMpGWB115 (Ishizaki et al. 2015) to generate the Mp*ACL5pro:Citrine-NLS* construct, in which a DNA fragment encoding a nuclear localized Citrine was translationally fused with the Mp*ACL5* fragment encoding the first eight amino acid residues of MpACL5. This vector was introduced into the *Rhizobium tumefaciens* GV3101 (pMP90) strain by electroporation and introduced into regenerating thalli of Tak-1 and Tak-2 as previously described (Kubota et al. 2013). Transformants were selected with 10 µg ml^−1^ hygromycin B and 100 µg ml^−1^ cefotaxime.

### Bacterial MpACL5 production

The aforementioned full-length MpACL5 fragment was excised by *Xba*I and *Hind*III and transferred into the pMal-c2 vector (New England BioLabs, Ipswich, MA, USA) to generate the pMal-MpACL5 construct. pMal-MpACL5, pMal-AtACL5 (Hanzawa et al. 2000) and the empty vector were introduced into *E. coli* DH5α. The transformed *E. coli* was cultured in 2 ml of Luria-Bertani medium supplemented with 2% glucose and 100 µg ml^−1^ ampicillin for 6 h and then cultured in the presence of 100 µM IPTG for 3 h to induce maltose binding protein-ACL5 fusion proteins. After centrifugation at 15,000 rpm for 1 min, precipitate was suspended with 800 µl of 5% perchloric acid, sonicated using a SONIFIER 250 (EMERSON BRANSON, Brookfield, CT, USA) for 1 min in constant mode output 2 on ice and centrifuged at 15,000 rpm at 4°C. A total of 400 µl of the supernatant was neutralized with 200 µl of 2 N NaOH and treated with 2 µl of benzoyl chloride at room temperature for 20 min. A total of 400 µl of a saturated sodium chloride solution was then added, followed by the addition of 400 µl of diethyl ether and vigorous mixing. After centrifugation at 4°C 3000*g* for 10 min, the organic layer was collected in 2-ml microtubes, evaporated and resuspended in 50 µl of methanol to make a polyamine extract. Polyamines were separated by using a reverse phase HPLC system equipped with TSKgel ODS-80Ts column (Toso, Tokyo, Japan) and detected by their UV absorbance at 254 nm (Tong et al. 2014).

### RT-qPCR

Total RNA was isolated from the thalli and gemma cups with gemma of 2-week-old Tak-1, gametangiophores of 1-month-old Tak-1 and Tak-2 and thalli of 2-week-old Tak-1, Tak-2 and Mp*acl5* mutants by NucleoSpin RNA Plant (Takara) or Monarch Total RNA Miniprep Kit (New England BioLabs) according to the manufacturer’s instruction. Gemma cups were separated from the thalli by a scalpel. These were immediately frozen in liquid N_2_ for subsequent RNA extraction. For each sample, 0.5 µg of total RNA was reverse transcribed to cDNA using ReverTra Ace reverse transcriptase (Toyobo, Osaka, Japan) according to the manufacturer’s protocol. Real-time PCR was performed using THUNDERBIRD Next SYBR qPCR Mix (Toyobo) and the primers, Mp*ACL5*-F (GGTGACACTGCACCAATCAC) and Mp*ACL5*-R (CTCCGGTGTGCAAGATTTTT) in the thermal cycle of 95°C 30 s—40 cycles of 95°C 5 s, 55°C 10 s and 72°C 30 s or using the KAPA SYBR FAST qPCR Kit (KAPA Biosystems) and gene-specific primers (**Supplementary Table S2**) in the thermal cycle of 95°C 2 min—40 cycles of 95°C 30 s, 55°C 30 s and 72°C 90 s on a thermal cycler Dice Real-Time System (Takara) according to the manufacturer’s method. Transcript levels of Mp*EF1α* or Mp*ACT7* were used as a reference for normalization (Kubota et al. 2014, Saint-Marcoux et al. 2015). Primers used in RT-qPCR are listed in **Supplementary Table S2**.

### RNA-seq

RNA-seq was conducted according to Yamaoka et al. (2018). Total RNA was isolated from 14-day-old thalli of the wild type and Mp*acl5* mutants grown on the half-strength B5 agar medium using NucleoSpin RNA Plant (Takara). The sequence libraries were generated using TruSeq RNA Sample Prep Kit (Illumina, San Diego, CA, USA) and sequenced in the Illumina HiSeq 1500 platform. Mapping of sequence reads and gene expression analysis were also conducted according to Yamaoka et al. (2018). Four biological replicates were used for RNA-seq analysis.

### Mutagenesis of Mp*ACL5*

For the CRISPR/Cas9 construct of Mp*ACL5*, complementary DNA oligos encoding 18-base target sequence of gRNA, gRNA-F (CTCG GCT GCT TGT GGT TCG AAG) and gRNA-R (AAAC CTT CGA ACC ACA AGC AGC), were annealed and cloned into the *Bsa*I site of an entry vector pMpEn_03 (Sugano et al. 2018). The MpU6-1pro:gRNA in pMpEn_03 was transferred into a binary vector pMpGE010 by LR reaction using Gateway LR Clonase II enzyme mix (Thermo Fisher Scientific). The resulting binary vector was introduced into the *Rhizobium tumefaciens* GV3101 (pMP90) strain by electroporation. The construct was introduced into wild-type sporelings (F1 spores produced by crossing Tak- 2 and Tak-1) by the *Agrobacterium*-mediated transformation method (Ishizaki et al. 2008). Plants were transferred to the half B5 agar medium containing 10 µg ml^−1^ hygromycin and 100 µg ml^−1^ cefotaxime. For the selection of mutants, genomic DNA of transformants was extracted and subjected to PCR by using a DNA polymerase KOD FX neo (Toyobo) and gene-specific primers to amplify DNA flanking the target sequence. The primers used for PCR are shown in **Supplementary Table S2**. By using the amplified DNA as a template, the sequencing reaction was performed using BigDye terminator ver.3.1 (Thermo Fisher Scientific) and a gene-specific primer (**Supplementary Table S2**). DNA sequence was analyzed using an ABI3500 Genetic Analyzer (Applied Biosystems, Waltham, MA, USA).

### Microscopy

Morphology of plants was observed by using a stereoscopic microscope S8APO0 equipped with a CCD camera DFC500 or a light microscope DM5000B equipped with DFC500 (Leica Microsystems, Wetzlar, Germany). The whole morphology of plants and gametangiophores was photographed using a single-lens reflex camera D5600 (Nikon, Tokyo Japan).

The thalli and reproductive organs were fixed overnight in a 1% aldehyde solution (glutaraldehyde 1%, phosphate buffer 5%) and substituted by ethanol while gradually increasing the ethanol concentration from 50% to 100%. Hundred percent ethanol was replaced with isoamyl acetate and dried in a critical point dryer JCPD-5 (JEOL, Tokyo, Japan). After drying, the samples were coated by gold evaporation using JFC-1200 Fine Coater (JEOL) and observed under a SEM JSM-6510LV (JEOL).

To analyze promoter activity of Mp*ACL5*, Mp*ACL5pro:Citrine-NLS* plants were grown on half-strength B5 medium under continuous white light. Gemma cups and gametangiophores were vertically hand-sectioned by razor blades. The intact gemmae and sectioned samples were placed on a glass slide with small aliquots of water, covered with a glass strip and observed under a FV1200 confocal laser scanning microscope (Evident, Tokyo, Japan) equipped with a high-sensitivity GaAsP detector and silicone oil immersion objective lenses [Evident, UPLSAPO30XS, 30×, numerical aperture (NA) = 1.05; UPLSAPO60XS2, 60×, NA = 1.3] or a FV3000 confocal laser scanning microscope (Evident) equipped with a high-sensitivity GaAsP detector, a water immersion objective lens (Evident, UPLSAPO60XW, 60×, NA = 1.2) and extended apochromat objective lenses [Evident, UPLXAPO10X (10×, NA = 0.4), UPLXAPO20X (20×, NA = 0.8), UPLXAPO40XO (40×, NA = 1.4, oil immersion lens)]. Silicone oil (SIL300CS, Evident) and immersion oil (F30CC, Evident) were used as immersion media for objective lenses. The samples were excited at 473 nm and 559 nm (laser diode). The emission was separated using a FV12-MHSY SDM560 filter (490–540 nm, 575–675 nm, Evident) in FV1200 and the multichannel TruSpectral detection in FV3000. The images were analyzed using ImageJ (National Institutes of Health, Bethesda, MD).

Proportions of S-phase cells were analyzed using Click-iT EdU Imaging Kits (Life Technologies, Carlsbad, CA) according to the manufacturer’s instruction. Gemmalings and thalli were transferred to the liquid 1/2 B5 medium containing 10 µM EdU and incubated for 1 h. Samples were fixed with a 3.7% formaldehyde solution in phosphate-buffered saline for 20 min. EdU incorporated into DNA was labeled with Alexa Fluor 488-azide. EdU-labeled cells were observed using a confocal laser scanning microscope FV3000 (Evident) as described earlier. The maximum *Z*-projection images were created using the ImageJ software.

### Measurement of chlorophyll

Chlorophyll was extracted from 50 mg of thalli in 1 ml of *N*,*N*-dimethylformamide at 4°C overnight in the dark and assayed as described (Porra et al. 1989).

## Supplementary Material

pcae002_Supp

## Data Availability

The data underlying this article will be shared on reasonable request to the corresponding authors.
