# Supplementary material for: Thermospermine Is an Evolutionarily Ancestral Phytohormone Required for Organ Development and Stress Responses in Marchantia Polymorpha
Source: Plant Cell Physiol. 2024 Jan 5;65(3):460–71. doi: 10.1093/pcp/pcae002 (PMC11020214; doi:10.1093/pcp/pcae002)
Supplement: pcae002_Supp [file pcae002_supp.zip › suppl_data/pcp-2023-e-00180-File011.pdf]

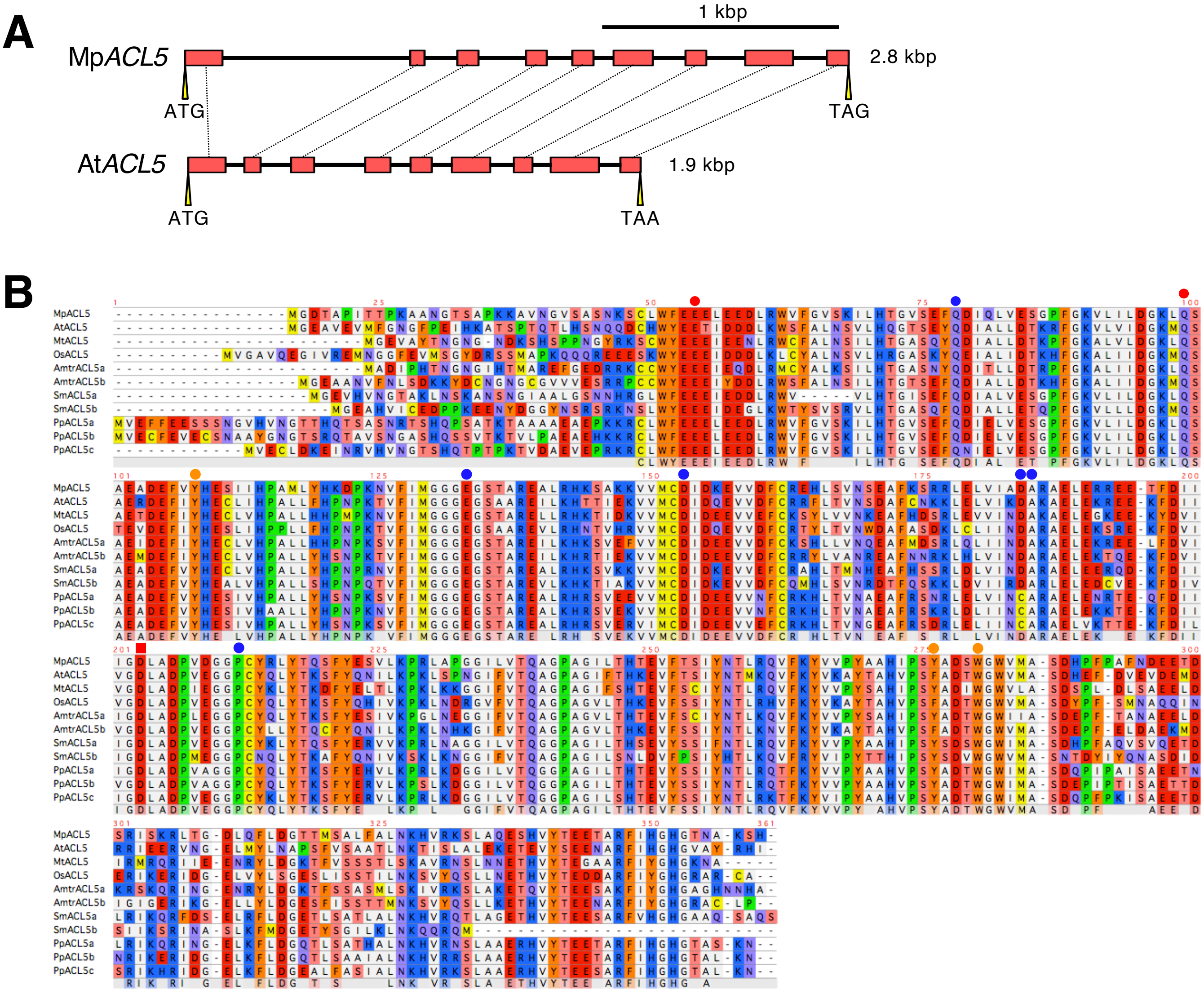

Supplementary Fig. S1 Structure of Mp*ACL5*. (A) Exon-intron structure of Mp*ACL5* and At*ACL5*. Protein coding regions are shown in red boxes. Bars indicate introns. (B) Sequence alignment of ACL5. Red circle and square, amino acid residue required for the binding of the amide of spermindine (aspartic acid marked by a red square is essential for the transfer of aminopropyl group from decarboxylated *S*-adenosylmethionine to spermidine); orange circle, aromatic amino acid residue required for the spermindine binding; blue circle, amino acid residue required for the binding of decarboxylated *S*-adenosylmethionine. Coloring of amino acids: red, acidic; blue, basic; orange, aromatic; purple, asparagine and glutamine; pink, serine and threonine; yellow, cysteine and methionine; green, proline. Amtr, *Amborella trichopoda*; At, *Arabidopsis thaliana*; Mp, *Marchantia polymorpha*; Mt, *Medicago truncatula*; Os, *Oryza sativa*; Pp, *Physcomitrium patens*; Sm, *Selaginella moellendorffi*.

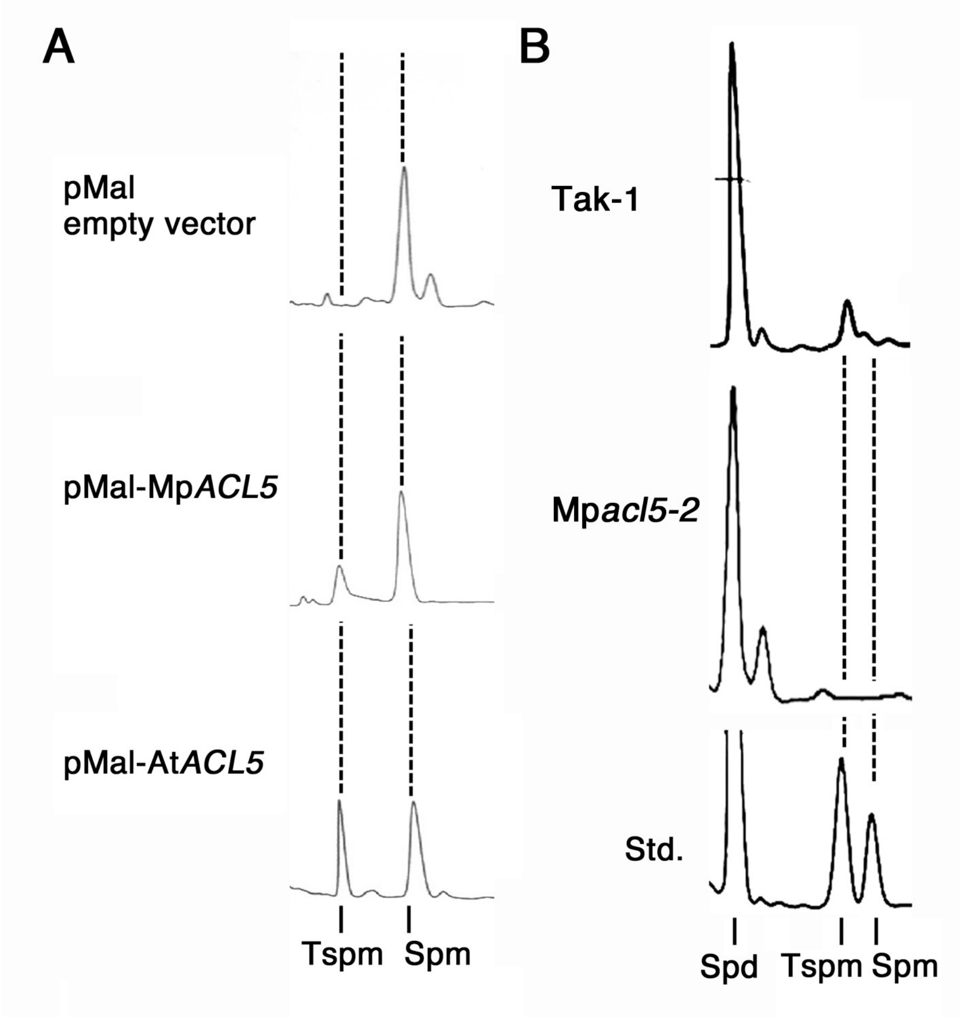

Supplementary Fig. S2 Detection of thermospermine by HPLC. (A) Detection of thermospermine in bacterial extracts. Polyamines were extracted from *E. coli* transformed with the empty pMal vector, pMal-Mp*ACL5*, or pMal-At*ACL5* and subjected to the HPLC analysis after benzoylation to distinguish thermospermine (Tspm) and spermine (Spm) according to Takano et al. (2012). (B) Detection of thermospermine in Tak-1 and Mp*acl5-2*. Polyamines were extracted from thallus and analyzed as in (A).

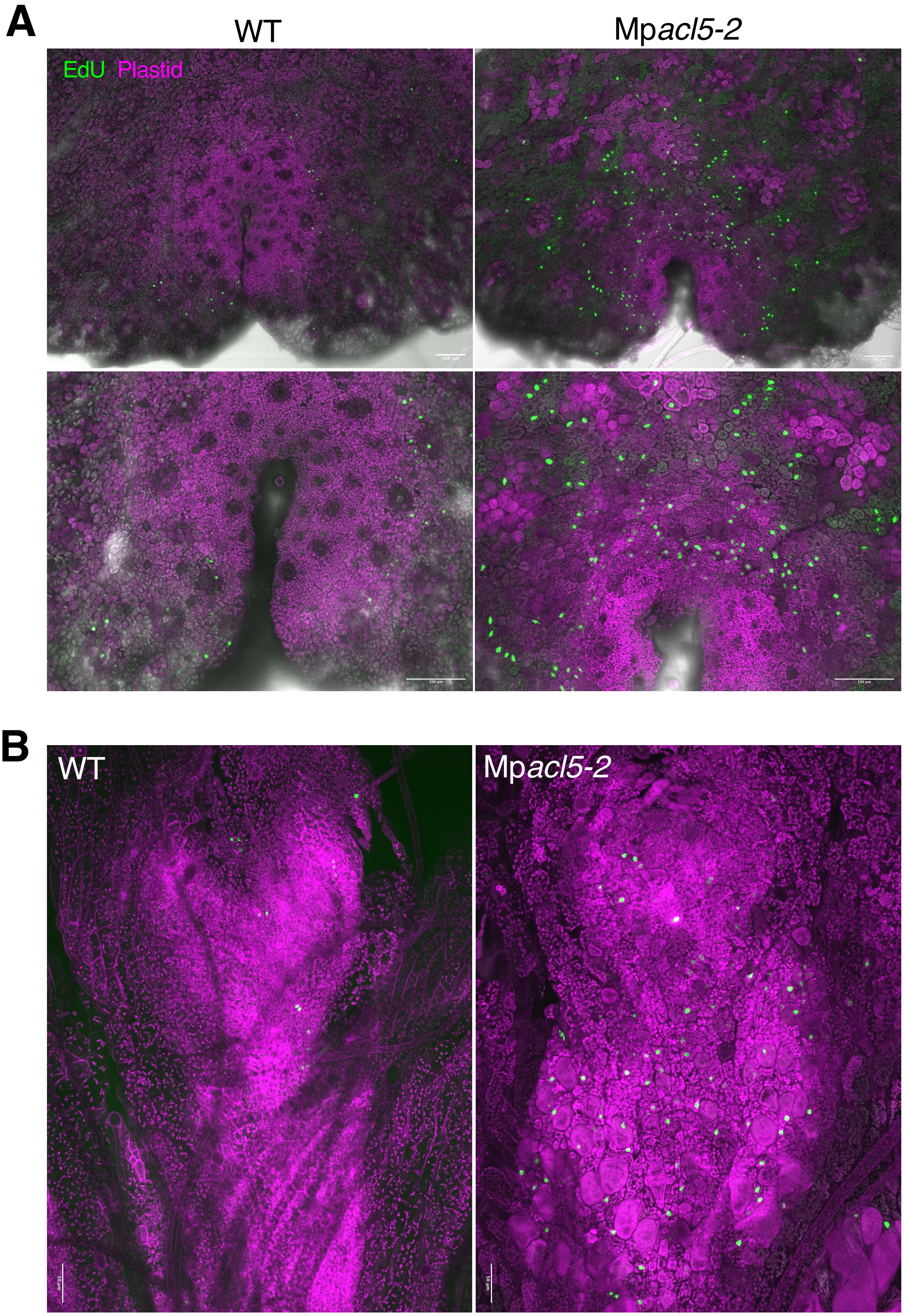

Supplementary Fig. S3 EdU staining. (A) Two-week-old thallus of the wild type (Tak-1) and Mp*acl5-2* stained by EdU. Bars = 100 µm. (B) Early reproductive organ in the four-week-old thallus of the wild type (Tak-1) and Mp*acl5-2*, which was irradiated by far red light for 8 days and stained by EdU. Bars = 50 µm. Green, EdU-positive nucleus; magenta, autofluorescence of plastids.

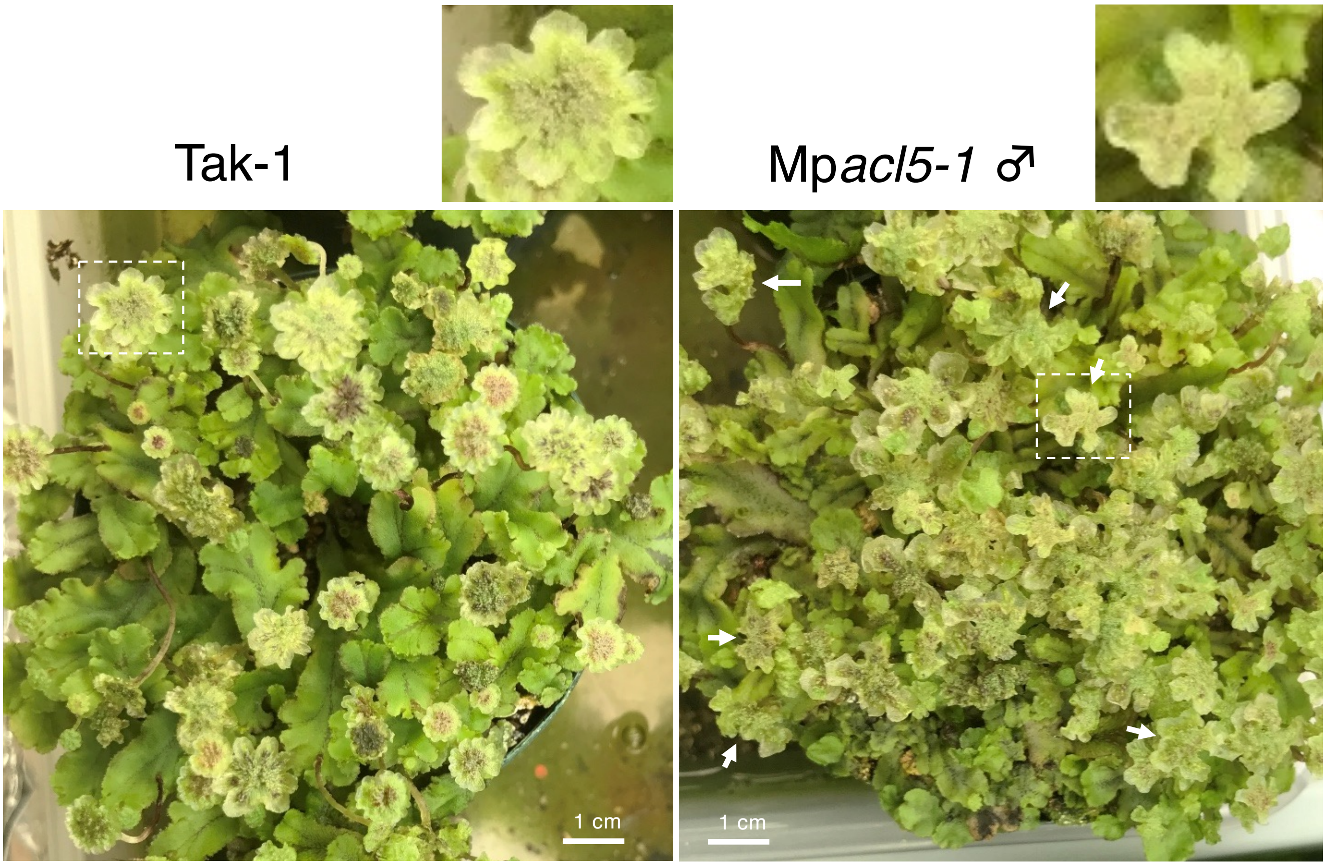

Supplementary Fig. S4. Fasciation phenotype of Mp*acl5* in the F1 progeny obtained by the crossing of Mp*acl5-1* with Tak-1. Top-view photographs of antheridiophores of Tak-1 and Mp*acl5-1* were shown. Arrows indicate obvious fasciation of antheridiophores.

A

```
GCCATCGCAGCTACCATGCGTACATCCATGGGCATAGTTCCAGGGGCCATACCATCATGAGAGGTGATTCAAA
GACATTACTGCGTTCGACCAGGGCTGGCTGGTTTTATGCAGGCCCAGCCTCCCCAATTGCCCTGTGCCCTCCC
AGTCAGATCGCGCTCGCCCGGAACCCGTCTCCGCTCCGCTCCTCCTTCCCCTCCTCTTCTCACGCGACCAACG
GAACCAACCTCTCGACGATTCGTTGGGCTTTTTTTATAAAGCAGTATCTTCAGCACTAACACCGCCTTTTTCC
CTGCAGCTTCGGGGTTCTCTCGATTGCAGGTGTCACTACTCGTAGATGGTTGCACACGCCGCCTTTTTTCACT
CTAGCAAATCATTGTATTGCTCGACGCCGGTCATCCTACACTACACTACTCCTTCACCTTTAAATGCGAACAA
CAGCAGGAAACACATGATTTCGTTCGCGGCGTCGCATTCACAGACTGACATTTTGTGACATACATCATTCTCG
CTGAAATCTGCTCAACGTTTCCAGCTGCCGTGTCGCCGCCCGTCCGGATTACTTTCGGACTGTAACATATCTC
TCGGGGCGTACGTGAAGACTGGAAATGTTCGAGGATTTGGATTTGGGTACAGCTTGTAAGAATTTTCAGAATC
TGGCCGCTGATTTAGTAGAGCGGTGGCAGTCCCAGAAACCGTGTACAGAGTCGGGACGTTTTAACTACATCAT
GTTTGATCAGAGCGGGGGAAAGGGGCGGATCTTCCAGCACCCTTCTCGCCTGCTGCACGAGTTCCCGACGAGC
TCTTCGTCGCCTCCGCAGCCTAACTCTCCAGACTTGTACCAGGACATGTCTCTCCAGGAGTTCGAGGACACTC
TTCTGAGGAACTCTGTGCAGGACAAATGCGATCCCCGAATGGCGGCCTGCCGAGAGACGGTGGCTTCGGCCTA
CGCTGCAGTCCAGCAGCAGCAGCAAGACAAAAACGACGATCAGATTCGCCATCAAAATCCGACAGCTTTGTTT
CCCGAGGAGGTTTACAGCGCATTTCAAGCGAGCGAGGAGGAATCCAGCCGAGCCGCAGGAGGAGGCTACCTGT
ACGAGGATGCCGACGACTTGGAAGCGCTGCTAGGATGCTCCGACGAGGAAGAGAGCATCACCGGCAATGCGCC
GAGCAGCGAATTGACCTGCCATTTCGGCCTTCAGGAGGACGACAACATCCGACCGTGCGAGCCTTCGTCGAGG
AAGAGGCCGTGCGTGTTGGACGAGGAGGTCGACTCGAAATTCAAGCGGCAGAGGTCGCAGGAGGAGGAGGAAG
AGGAAGTCGAGGACATTTGCATCGGCACCTGCTCGTCGAGTGTCTCGGTGGTCGATGCCTTCAGTGGCGATTC
CCTGGGTCCCCCTTGCGCCCTGGAGAGCGAAGCAGACGACTGTCTGGACGAGTCTGCGCTCGAGCTGCAAATG
CTGTGCGACGACTTCGGCTACGAGATGCCTGAGGAGCAACAATGTAGCTCATGCAGCAAGGAGGACGGAGACG
AAGGCGACTCGATCTCATCCGGCTCGGCCAGGAAGTCTAGGCGAGATAAGATTAAGAAGACCGTGAAGCTCTT
GAGAAACATCATTCCAGGCGGAGATTGCATGGACACGGCCATCGTTCTGGACGAGGCTATACACTATGTGAAG
TTGCTCCAATTGCAGGTTCAGACTTTGGAGGCCAAGAGGCTCGTCGAGAGAGTTCGGTGACCCTTGGGGCTCT
TATCTGCTCGAATCTTCATCCTCTTCCTACCTAATTAGTCTTATTTCTCGGCGAGGGTTTAGCCTAGTGAACG
CACGCCCCGAATATAGAGAGGCTCATACTCATCTCCCCGCTTTGATCACTACACCCTCCCCCACCCTTAGGAA
TCTTG
```

B

```
>AtSAC51     MVCQSPGKTRFRGLKYE-TGNANESTIVVRVIECYQPMDNCQAEYFRLLLKPVT
>AtSACL1     MVSQSAGQTRFRTFKYENNGDSSRPTIVVRVIACFQPMDNCQAEYFRHILKPVT
>AtSACL2     MVCQSAGQTRFRTLKHE-HGITGN--IVVRVIACFQPLQDCQAEYFRQLLKPVT
>AtSACL3     M-CIVGNKKGNRSLK-EI-GTFMMTTCF---IANYQSVQVCQAEYFRQLLKPVT
>SmSAC51     MMGQSAAGKSVGASPRKGSTLAAHPVSTSSIIACFEPLHERQAIYFRSMLKPIT
>MpBHLH42-1 MVAHAAFFHSSKSLYCSTPVILHYTTPSPLNANNSRKHMISFAASHSQTDIL
>MpBHLH42-2 MRGDSKTLLRSTRAGWFYAGPASPIALCPPSQIALARNPSPLRSSFPSSSHATNGTNLSTIRWAFFIKQYLQH
>MpBHLH42-3 MQAQPPQLPCALPVRSRSPGTRLRSAPPSPPLLTRPTEPTSRRFVGLFL
```

C

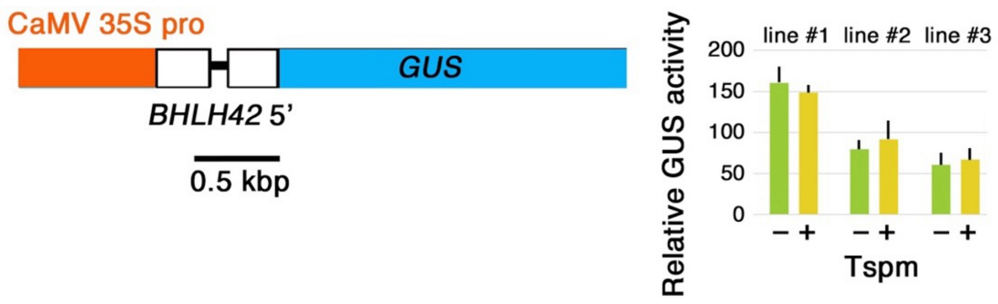

Supplementary Fig. S5 The closest homolog of *SAC51* in *M. polymorpha*, Mp*BHLH42***,** is not responsive to thermospermine. (A) The full-length cDNA sequence of Mp*BHLH42*. Start codons in the 5' leader region are boxed. Main coding sequence is shown in red letters. (B) Alignment of peptide sequences encoded by uORFs conserved in the *SAC51* family in *Arabidopsis* and *Selaginella moellendorffii* (SmSAC51) with those present in Mp*BHLH42*. (C) The Mp*BHLH42* 5' leader-GUS fusion construct and the GUS activity in transgenic *Arabidopsis* seedlings carrying the construct. The 5' leader region was amplified from genomic DNA by PCR with primers, FXba, TCT AGAGCCATCGCAGCTACCATGC, and RBam, GGAT CCCAAATCCAAATCCTCGAAC, and inserted as a XbaI-BamHI fragment into pBI121. Activities in three independent transgenic lines are shown. Ten-day-old seedlings were incubated with 0.1 mM thermospermine for 24 h. Bars indicate SD (n=5).
