# Supplementary material for: Thermospermine Is an Evolutionarily Ancestral Phytohormone Required for Organ Development and Stress Responses in Marchantia Polymorpha
Source: Plant Cell Physiol. 2024 Jan 5;65(3):460–71. doi: 10.1093/pcp/pcae002 (PMC11020214; doi:10.1093/pcp/pcae002)
Supplement: pcae002_Supp [file pcae002_supp.zip › suppl_data/pcp-2023-e-00180-File012.pdf]

Supplementary Table S1. Genes upregulated or downregulated in Mp*acl5* mutants

Gene number	Gene ID	Gene name	Fold change (Mp*acl5* /WT, log2)	Annotation
Upregulated genes				
Mp2g00930	Mapoly0028s0058	MpBHLH4	7.83	bHLH-MYC and R2R3-MYB
Mp1g09210	Mapoly0036s0157		7.81	Unknown
Mp5g22160	Mapoly0166s0010	MpERF21	7.71	Ethylene-responsive transcription factor
Mp5g07490	Mapoly0127s0035		7.53	Unknown
Mp7g07900	Mapoly0076s0004		6.90	HSP20
Mp7g07910	Mapoly0076s0003		6.84	HSP20
Mp6g00400	Mapoly0104s0026		6.22	Unknown
Mp8g13020	Mapoly0083s0019		6.22	Unknown
Mp2g00890	Mapoly0028s0062	MpBHLH2	6.02	bHLH-MYC and R2R3-MYB
Mp7g02470	Mapoly0088s0039		5.90	Protein phosphatase inhibitor
Mp7g06480	Mapoly0057s0019		5.40	HSP17.6
Mp5g23140	Mapoly0010s0142		5.37	HSP17.6
Mp7g18870	Mapoly0067s0090		5.19	Methionyl-tRNA synthase
Mp2g20590	Mapoly0195s0009		5.08	Unknown
Mp2g16410	Mapoly0122s0023		5.01	Nucleic acid binding protein
Mp2g01700	Mapoly0983s0001		4.99	Copper chaperone, Copper transport protein
Mp1g07890	Mapoly0036s0033		4.95	Unknown
Mp6g11710	Mapoly0223s0001	MpMTPSL22	4.84	Terpene synthase family 2, Farnesyl diphosphate synthase
Mp2g20110	Mapoly0055s0038	MpCQC	4.78	Terpenoid cyclases, Oxidosqualene-lanosterol cyclase related
Downregulated genes				
Mp4g19900	Mapoly0126s0004		-5.18	Phosphate transporter
Mp8g16710	Mapoly0030s0004		-5.10	Unknown
Mp2g18440	Mapoly1958s0001	MpCYP829-like12	-4.83	Cytochrome P450, 93A3-like
Mp2g07390	Mapoly0015s0026	MpOMT8	-3.99	Caffeic acid 3-O-methyltransferase like
Mp8g10910	Mapoly0008s0131	MpUGT36	-3.95	UDP-glucuronosyl and UDP-glucosyl transferase
Mp3g01440	Mapoly0007s0137		-3.85	4Fe-4S ferredoxin、photosystem I iron-sulfur center
Mp7g07360	Mapoly0076s0058		-3.84	Unknown
Mp7g05640	Mapoly0057s0107		-3.79	Actin-binding LIM Zn-finger protein Limatin like
Mp5g07800	Mapoly0127s0004		-3.65	GDSL-like lipase/acylhydrolase、
Mp3g18480	Mapoly0225s0001		-3.58	Tornado1 like protein
Mp7g06460	Mapoly0057s0024		-3.57	Unknown
Mp8g09900	Mapoly0008s0232		-3.42	Haem peroxidase superfamily
Mp3g07190	Mapoly0006s0192		-3.36	Calcineurin-like phosphoesterase
Mp5g20300	Mapoly0058s0007		-3.36	photosystem II oxygen-evolving enhancer protein 2, Mog1p/PsbP-like
Mp5g14670	Mapoly0032s0159		-3.35	Cinnamyl alcohol dehydrogenase like
Mp8g16290	Mapoly0154s0035	MpMLS5	-3.33	Malate synthase
Mp3g24170	Mapoly0121s0007	MpDIR45	-3.29	Dirigent protein
Mp4g17620	Mapoly0041s0044		-3.27	U1 snRNP-specific protein, DMP3(DUF679 domain membrane protein 3)
Mp8g12190	Mapoly0083s0098		-3.27	Serine/threonine phosphatase 2C containing leucine-rich repeats
