# Supplementary material for: Thermospermine Is an Evolutionarily Ancestral Phytohormone Required for Organ Development and Stress Responses in Marchantia Polymorpha
Source: Plant Cell Physiol. 2024 Jan 5;65(3):460–71. doi: 10.1093/pcp/pcae002 (PMC11020214; doi:10.1093/pcp/pcae002)
Supplement: pcae002_Supp [file pcae002_supp.zip › suppl_data/pcp-2023-e-00180-File013.pdf]

Supplementary Table S2. Primer sequences used for RT-PCR and genotyping of Mp*acl5* .

Gene number	Primer sequence
Mp7g06480 (HSP)	F: CAGATCTTCCTGGCATGAAG
	R: TGTCTTCTTGGACTCTTCGG
Mp7g07900 (HSP)	F: GCAGATCTTCCTGGCATGAA
	R: CTTGGACTCTTCGGTCTTGG
Mp1g09210	F: CCGGAATCATTCGATGATAAC
	R: TGCAGCTGCTGAGCTGAATT
Mp5g07800	F: TCTTACGGATTCAATCCAGC
	R: CAAGTTTCTTGAACTGGGAC
Mp2g07390 (MpOMT8)	F: GATGCTGTATTCATGAAGTGG
	R: CAAGTTTCTTGAACTGGGAC
Mp8g16710	F: AATCCACTGCACGACGCTCT
	R: GGACCGAGTTTCTGACTTCA
Mp6g11010 (MpACT7)	F: AGGCATCTGGTATCCACGAG
	R: ACATGGTCGTTCCTCCAGAC
Mp8g03070 (MpACL5)	editF: CAAGAGCTGCTTGTGGTTCG
	ex4R: CAAGAGTCGGCGTACGAT
